# Supplementary material for: pH-Sensitive Ratiometric Fluorescent Probe for Evaluation of Tumor Treatments
Source: Materials (Basel). 2019 May 18;12(10):1632. doi: 10.3390/ma12101632 (PMC6566363; doi:10.3390/ma12101632)
Supplement: Supplementary file 1 [file materials-12-01632-s001.pdf]

materials

Supplementary Materials

pH-Sensitive Ratiometric Fluorescent Probe for Evaluation of Tumor Treatments

Peisen Zhang [1,2], Junli Meng [1,2], Yingying Li [1,2], Zihua Wang [1,]* and Yi Hou [1,3,]*

1 Key Laboratory of Colloid, Interface and Chemical Thermodynamics, Institute of Chemistry, Chinese Academy of Sciences, Beijing 100190, China; zhangps@iccas.ac.cn (P.Z.); mengjl@iccas.ac.cn (J.M.); liyingying@iccas.ac.cn (Y.L.)
2 School of Chemistry and Chemical Engineering, University of Chinese Academy of Sciences, Beijing 100049, China
3 College of Life Science and Technology, Beijing University of Chemical Technology, Beijing 100029, China
* Correspondence: wangzh@iccas.ac.cn (Z.W.); houyi@iccas.ac.cn (Y.H.); Tel.: +86-10-8236-2540 (Y.H.)

Received: 30 April 2019; Accepted: 16 May 2019; Published: date

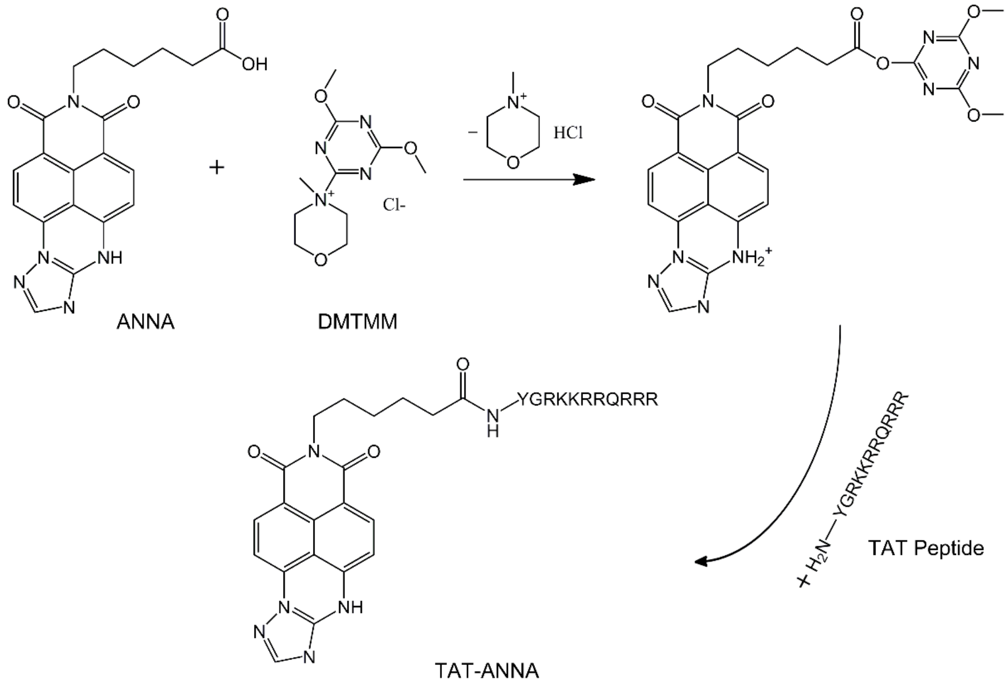

Figure S1. Synthesis of TAT-ANNA.

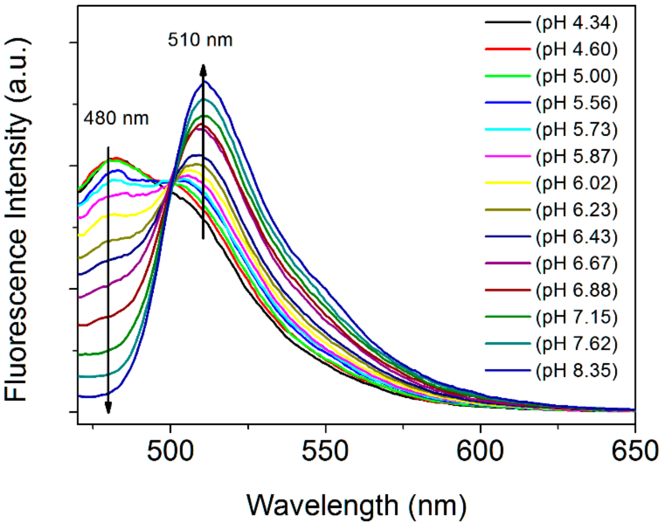

Figure S2. Fluorescence spectra of ANNA dye.
